# Capacitive Humidity Sensor Based on Carbon Black/Polyimide Composites

**DOI:** 10.3390/s21061974

**Published:** 2021-03-11

**Authors:** Jihong Kim, Jang-Hoon Cho, Hyung-Man Lee, Sung-Min Hong

**Affiliations:** 1 Department of Electrical Engineering, Yeungnam University, Gyeongsan 38541, Korea; jihongkim@yu.ac.kr; 2 Smart Sensor Research Center, Korea Electronics Technology Institute, Seongnam 13509, Korea; speed3064@keti.re.kr (J.-H.C.); lhm0703@keti.re.kr (H.-M.L.)

**Keywords:** humidity sensor, capacitive humidity sensor, polyimide, carbon black

## Abstract

A novel capacitive humidity sensor based on carbon black/polyimide composites is presented in this paper. The details of the fabrication, sensor characteristics, and effect of the carbon black additive are described. It was confirmed that the polyimide composite filled with a tiny amount of carbon black was suitable for a humidity sensing dielectric. The humidity sensors with three different dielectrics, which were pure polyimide, 0.01 wt% carbon black/polyimide, and 0.05 wt% carbon black/polyimide, were fabricated by a micro-electro-mechanical-system (MEMS) process. As the amount of the carbon black additive increased, the sensitivity of the humidity sensor increased. The humidity sensor with 0.05 wt% of carbon black had a much higher sensitivity of 15.21% (20–80% RH, 0.2535%/% RH) than that of the sensor with pure polyimide, which was 9.73% (0.1622%/% RH). The addition of carbon black also led to an enhancement in the hysteresis and response speed. The hysteresis of the humidity sensor decreased from 2.17 to 1.80% when increasing the amount of the carbon black additive. The response speed of the humidity sensor with 0.05 wt% of carbon black was measured to be ~10% faster than that of the sensor with pure polyimide. The long-term stability of the humidity sensors was demonstrated as well.

## 1. Introduction

Measurement of humidity, the amount of water vapor in air, is very important in many applications, including manufacturing, agriculture, climatology, and biomedical industry [1]. There are two ordinary measurements of humidity: absolute and relative humidity. Absolute humidity means a mass of water content in a unit volume of air. On the other hand, relative humidity describes a ratio of the amount of water vapor to that of the saturated air at a given temperature. Since controlling relative humidity level is generally preferable, humidity sensors usually measure relative humidity rather than absolute humidity [2].

For accurate measurement of relative humidity, various humidity sensors with different sensing mechanisms have been suggested such as capacitive, resistive, electromagnetic, gravimetric, and optical measurements [3,4]. Among them, capacitive type humidity sensors are preferable and dominate the market due to several advantages such as low power consumption, high sensitivity, and linear response [5]. The typical structure of capacitive humidity sensors is composed of two (top and bottom) electrodes and a humidity-sensitive dielectric material located between them, which is called a parallel plate capacitor structure. In this structure, the capacitance of the dielectric material can be measured in relation to the alternating current and the applied electric field between the two electrodes. The other popular structure is an interdigitated type where a pair of interdigitated comb-shaped electrodes (IDEs) is employed on the same plane. The IDEs create fringing electric fields, and therefore the capacitance of the dielectric material placed on (or beneath) the IDEs can be measured [6].

The dielectric constant of water vapor is around 80, and a humidity-sensitive dielectric material generally has a much lower dielectric constant. Therefore, absorption of water vapor by the dielectric results in an increase in the capacitance of humidity sensors [7]. Diverse materials have been extensively studied for the dielectric of humidity sensors, and they can be roughly categorized into two types: polymer-based and ceramic-based materials. Between them, polymer-based materials have been widely used owing to their low cost, easy fabrication process, and good stability [8]. In recent years, there have been efforts to make polymer composites with diverse carbon fillers for humidity sensor applications. In particular, carbon nanomaterials are widely used as reinforcing fillers for the polymer composites. However, most of the works have focused on modifying the conductance of the polymer composites in order to enhance the characteristics of resistive-type humidity sensors [9,10,11,12,13]. As far as we know, there have been few reports on capacitive humidity sensors based on carbon-polymer composites.

To develop an improved capacitive humidity sensor, we suggest a carbon-polymer composite, which consists of polyimide and carbon black. Polyimide is suitable as a humidity sensing material because of its linear and large response to humidity and compatibility with the complementary metal-oxide-semiconductor (CMOS) process [14]. In addition, a commercially available and photosensitive polyimide provides the advantage of an easy and low-cost fabrication process. Many researchers have tried to use polyimide for capacitive humidity sensors with various approaches. Changing the surface morphology of polyimide by plasma treatment is an efficient method to improve the performance of humidity sensors [15,16,17,18,19,20]. Applying novel electrodes or sensor structure design for polyimide dielectrics is also an effective way to optimize humidity sensors [21,22,23,24,25,26,27]. Integration of a heater element can help to improve the hysteresis and recovery of polyimide sensing layers [28,29,30,31,32]. However, research on synthesis of carbon-polyimide composites for capacitive humidity sensors has not been reported yet. Carbon black is the most widely used carbon nanomaterial since it has large surface area, and commercial products with different sizes can be obtained easily at low cost [33]. Carbon black/polyimide composites could be a strong candidate as a sensing dielectric material for capacitive humidity sensors. In the composite, moisture could be stored at the interface between the carbon black additive and the polyimide, resulting in an increase in water uptake. However, as is well known, an excessive addition of carbon black in polyimide would give rise to an increase in the conductance of the composite. This leads to the occurrence of considerable leakage current through the dielectric, which means the capacitor breaks down. In order to prevent this undesirable phenomenon, the amount of the carbon black additive must be controlled precisely.

In this paper, we report for the first time a novel capacitive humidity sensor based on a carbon black/polyimide composite. The humidity sensor is designed to have a parallel plate capacitor structure and be fabricated by the micro-electro-mechanical-system (MEMS) process. The characteristics of the developed humidity sensor such as sensitivity, hysteresis, dynamic response, and long-term stability are evaluated accurately by a computer-controlled measurement system. The comparison with the humidity sensor made of pure polyimide is also provided.

## 2. Experimental Details

### 2.1. Preparation of Carbon Black/Polyimide Composite

In order to synthesize the carbon black/polyimide composite, a photodefineable polyimide solution (HD-4100, HD Microsystems, Parlin, NJ, USA) and carbon black nanopowders with a diameter of 100 nm (Nanoshel, Wilmington, DE, USA) were employed. As mentioned above, in this work, the key parameter to achieve the novel sensing dielectric for the capacitive humidity sensor is the amount of the carbon black additive. Tiny amounts of carbon black were added to the polyimide solution to prevent an increase in the conductance of the composite. To verify the effect of the amount of carbon black on the sensing characteristics, three samples with different amounts of carbon black were prepared, which were 0 (pure polyimide), 0.01, and 0.05 wt%, respectively. Undesirable side effects of the carbon black additive on photolithography of the composite can also be avoided with these small amounts. After sealing, the carbon black additive in the composite solution was dispersed by using an ultrasonic bath for 60 h.

### 2.2. Sensor Design

**Figure 1:**
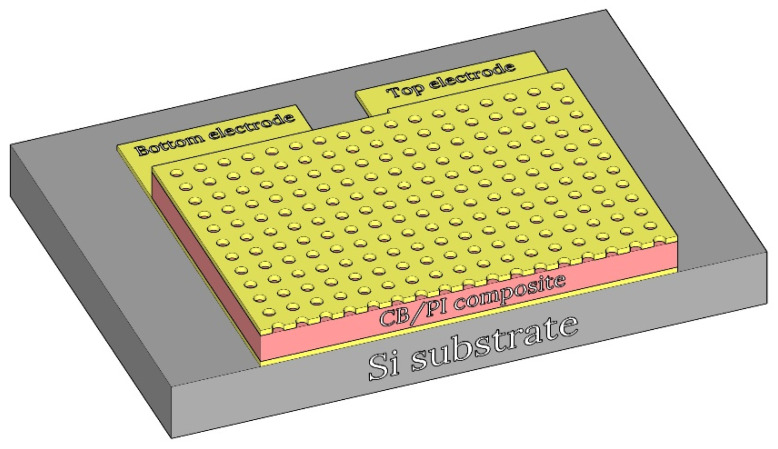
Simplified cross-section of the sensor design.

The humidity sensor was designed to have a parallel plate structure for the measurement of the capacitance. The simplified cross-section of the sensor design is shown in Figure 1. The sensor is comprised of two electrodes (top and bottom), and, between them, a carbon black/polyimide composite film is located as a sensing dielectric layer. The variation of the capacitance of the composite layer with relative humidity can be measured by using the two electrodes. The top electrode has many holes with a radius of 10 μm through which the composite film is exposed to atmosphere, resulting in an increase in the contact area with water vapor. The size of the sensor is 2 mm by 2 mm, which allows the sensor to be possibly installed in any portable equipment.

### 2.3. Fabrication of the Sensor

The humidity sensor was fabricated by the MEMS process. For insulation, a 4-inch silicon wafer on which a 300 nm-thick SiO_2_ layer was grown was used as a substrate. The bottom electrode with a 300 nm-thick Au film was formed on the substrate by e-beam evaporation and photolithography. A 300 nm-thick SiO_2_ passivation layer was also deposited and patterned to expose the active region of the underlying bottom electrode. After that, the prepared carbon black/polyimide composite solution was spin-coated for the humidity sensing layer. The coated composite film was patterned by photolithography and was cured in a vacuum curing chamber. After the curing process, the 3 μm-thick carbon black/polyimide composite sensing layer was successfully obtained. At last, for the top electrode, a 300 nm-thick Au film was deposited by sputtering and patterned to have the hole shapes as described in Section 2.2.

### 2.4. Measurement System

**Figure 2:**
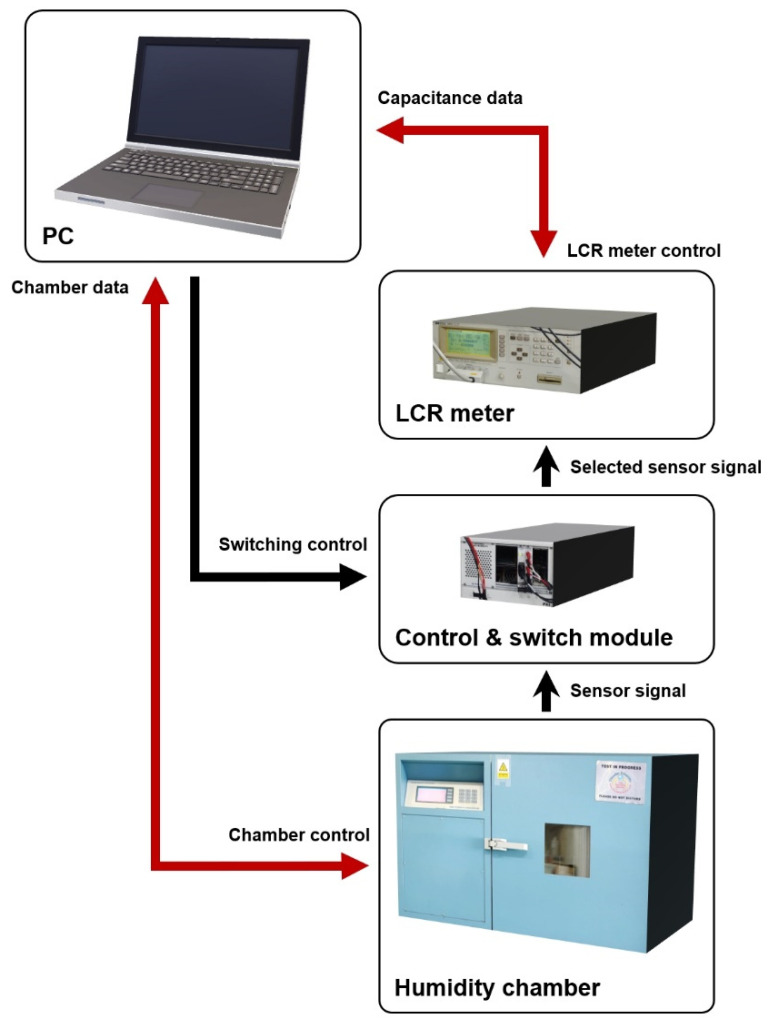
Schematic diagram of the measurement system.

The measurement system was fully automated and capable of multiple measurements up to 32 samples. The fabricated humidity sensors were wire-bonded to printed circuit boards (PCBs), and the PCBs were connected to a control and switch module (SCXI series, National Instruments, Austin, TX, USA). The signal from a selected sensor by the switch was transferred to a precision LCR meter (4284A, Hewlett-Packard, Palo Alto, CA, USA). To control relative humidity, the PCBs were placed in a humidity generating chamber (model 2500, Thunder scientific corporation, Albuquerque, NM, USA), and the variation of the capacitance of the humidity sensors was measured by using the LCR meter. When measuring the capacitance at each humidity level, to minimize the effect of possible fluctuations of the humidity chamber, ten data points were collected, and the average value was taken. In addition, because capacitance can arise from not only the sensors but also the PCBs, measurement cables, and air, one PCB on which the humidity sensor was not mounted was measured in the chamber to clarify the parasitic element. After that, the parasitic capacitance was subtracted from the measurement results of the actual humidity sensors. Every measurement was repeated at least ten times for a single device to check the reproducibility, and ten devices randomly picked from the same wafer were also verified to eliminate any possibility of errors from fabrication non-uniformity. The schematic of the measurement system is shown in Figure 2.

## 3. Results and Discussion

### 3.1. Imaging

**Figure 3:**
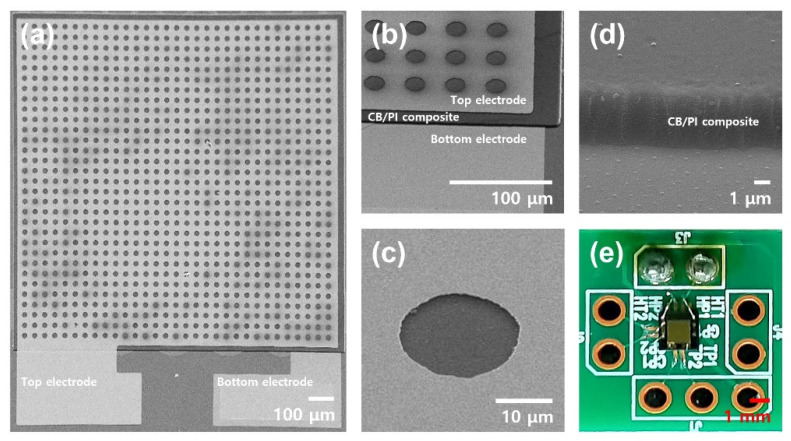
SEM images of the (**a**) fabricated humidity sensor, (**b**) top electrode/sensing dielectric layer/bottom electrode stack, (**c**) hole pattern in the top electrode, and (**d**) carbon black/polyimide composite after photolithography patterning. The humidity sensor mounted on a PCB is shown in (**e**).

Figure 3a shows the image of the fabricated humidity sensor acquired using a scanning electron microscope (SEM). The magnified SEM images of the top electrode/sensing dielectric layer/bottom electrode stack and the hole pattern in the top electrode are also shown in Figure 3b,c, respectively. It can be seen from the images that the whole fabrication process of the humidity sensor was performed flawlessly. Figure 3d shows the SEM image of the pattern edge of the carbon black (0.05 wt%)/polyimide composite. As expected, it is confirmed that the carbon black/polyimide composite layer was patterned successfully without any problem arising from the carbon black additive. Figure 3e shows the humidity sensor mounted and wire-bonded to a PCB that is ready to be connected to the measurement system.

### 3.2. Sensor Characteristics

#### 3.2.1. Sensitivity

**Figure 4:**
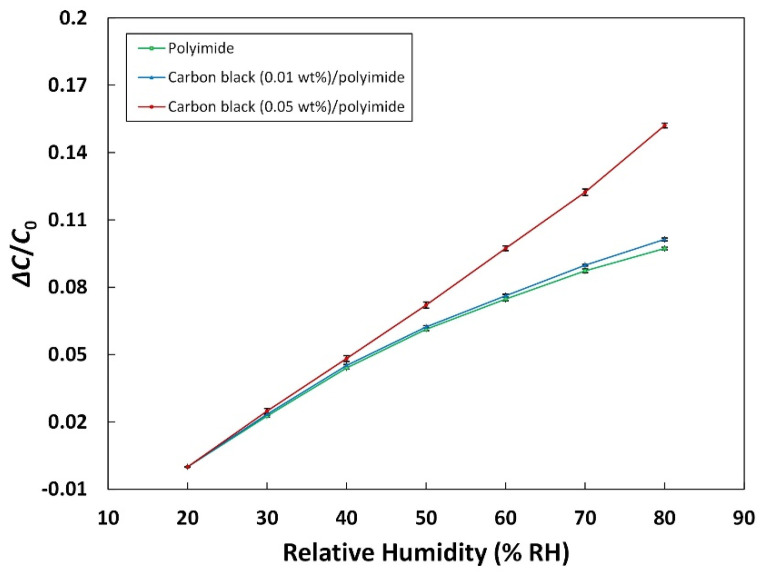
Sensitivity of the humidity sensors.

The sensitivity of the humidity sensor with a good linearity can be expressed as follows:

(1) $$S=\frac{C-C_{0}}{C_{0}}$$

where $C_{0}$ represents the initial capacitance of the sensor measured at 20% RH humidity level. Figure 4 shows the sensitivity of the humidity sensors with the different amounts of carbon black as a function of relative humidity. The error bars stand for the standard deviation calculated from ten times repetition. It can be confirmed that all three different sensors exhibit good linearity. The linearity is represented by the determination coefficient R^2^ from the sensor output and the regression linear curve. The calculated coefficients of the sensors with 0, 0.01, and 0.05 wt% of carbon black are 0.979, 0.982, and 0.999, respectively. The sensor with 0.01 wt% of carbon black has a slightly higher sensitivity of 10.13% (20–80% RH, 0.1688%/% RH) than that of the sensor made of pure polyimide, which is 9.73% (0.1622%/% RH). The amount of 0.01 wt% of carbon black seems not enough to significantly change the dielectric property of the composite, which results in little difference in the sensitivity. On the other hand, the sensor with 0.05 wt% of carbon black shows a sensitivity of 15.21% (0.2535%/% RH), which is much higher than that of the sensor with pure polyimide. It is known that moisture in the polymer composite can be stored at the interfacial space between the additives and the polymer [34]. The increase in the sensitivity of the humidity sensor could also be explained by the space at the interface between the carbon black additive and the polyimide matrix that serves as a site for moisture storage. Because of the large surface area of the carbon black nanoparticles, the area of the interface is also large, resulting in the increase in the water contact area, adsorption, and uptake. However, it was confirmed that the addition of carbon black more than 0.05 wt% (not shown here) rather deteriorated the performance of the humidity sensor. This phenomenon can be explained by obstructing the diffusion of water into the composite by excessive amounts of the carbon black additive. The major mechanism of humidity sensors using polymer materials is usually based on water diffusion into the polymer matrix. Kwon et al. tried to add large amounts of carbon black into the polyimide to enhance the mechanical properties as a passivation film. They proved that the addition of large amounts of carbon black can decrease the water diffusivity into polyimide by using a dynamic vapor sorption intrinsic analyzer. [35]. Based on all these results, it can be concluded that the amount of the carbon black additive has a great effect on the property of the capacitive humidity sensor, and the optimal proportion should be figured out. Furthermore, when considering the change in the conductance of the composite that can lead to a breakdown of the capacitor, the precise control of the amount of carbon black is extremely important. In our case, because of the little amount of the additive, a very high resistance (out of range) was observed for every sensor, indicating no breakdown of the dielectric.

#### 3.2.2. Hysteresis

**Figure 5:**
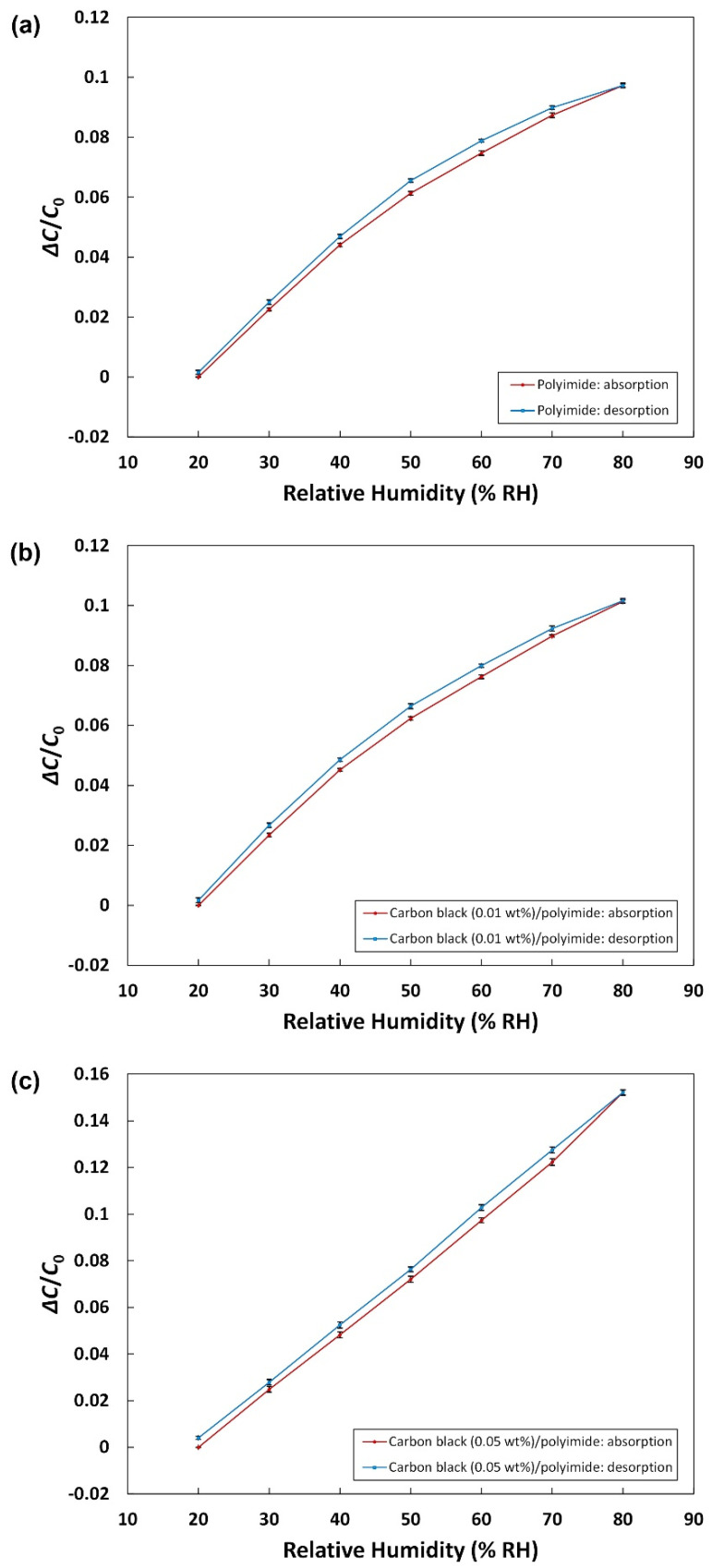
Hysteresis characteristics of the humidity sensors with (**a**) pure polyimide, (**b**) carbon black (0.01 wt%)/polyimide, and (**c**) carbon black (0.05 wt%)/polyimide.

The hysteresis characteristic of humidity sensors when water is absorbed and desorbed is very important to measure humidity accurately. The capacitance of the fabricated sensor was measured during a cycle of water absorption (from low to high ambient relative humidity) and desorption (from high to low ambient relative humidity). The hysteresis error can be described as follows:

(2) $$H_{E}=\pm \frac{\Delta H_{\max}}{2FS}$$

where $\Delta H_{\max}$ is the maximum difference in output during the absorption and desorption, and FS denotes the full-scale output. Figure 5a–c show the hysteresis characteristics of the humidity sensors with 0, 0.01, and 0.05 wt% of carbon black, respectively. In Figure 5a–c, the maximum absolute values of the hysteresis error are calculated to be 2.17, 2.02, and 1.80%. Although all three different sensors present quite low values, the addition of carbon black in the polyimide made the hysteresis characteristic better. The hysteresis of humidity sensors based on polymers is thought to be due to the formation of clusters of sorbed water molecules [36]. In the carbon black/polyimide composite, the carbon black particles with a large surface area give rise to an increase in the contact area with water molecules, which can make water molecules more dispersed without forming clusters and easier to be desorbed. This seems to be the reason for the improved hysteresis of the humidity sensor with the carbon black/polyimide composite.

#### 3.2.3. Dynamic Response

**Figure 6:**
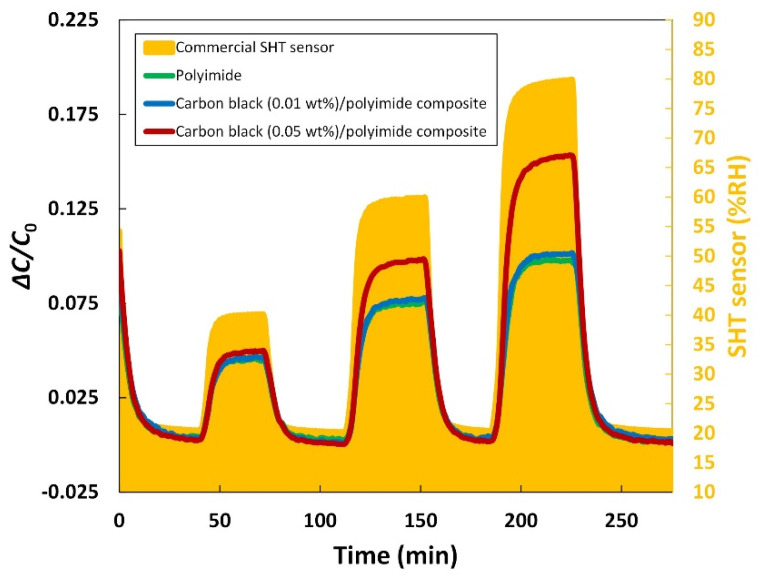
Dynamic response of the humidity sensors.

To study the dynamic response of the humidity sensor, the humidity level in the chamber was ramped up and down between 20% RH and three different higher levels, which are 40, 60, and 80% RH, respectively. Figure 6 shows the dynamic response of the humidity sensors. It can be observed that all three humidity sensors respond properly to the change in the humidity. In terms of the response speed, however, the addition of carbon black made a difference. When the humidity was changed between 20% RH and 80% RH, the response time, which was defined as a time required to reach 90% of the entire output change, of the humidity sensor with 0.01 wt% of carbon black was not significantly improved compared to that of the sensor with pure polyimide. On the other hand, the sensor with 0.05 wt% of carbon black had a response speed ~10% faster than that of the others. This result can also be explained by the role of carbon black in increasing the contact area and desorption speed as mentioned above. For comparison, a widely used commercial humidity sensor (SHT3x, Sensirion) was measured under the same condition. As can be seen in Figure 6, all of the developed humidity sensors have a relatively slower response than the commercial sensor. The humidity sensor with 0.05 wt% of carbon black showed ~1.3 times slower response during the humidity variation between 20% RH and 80% RH. Nevertheless, this performance is still comparable and considered as acceptable for use in humidity measurement, taking into account the excellent performance of the commercial sensor that has been already optimized.

#### 3.2.4. Long-Term Stability

**Figure 7:**
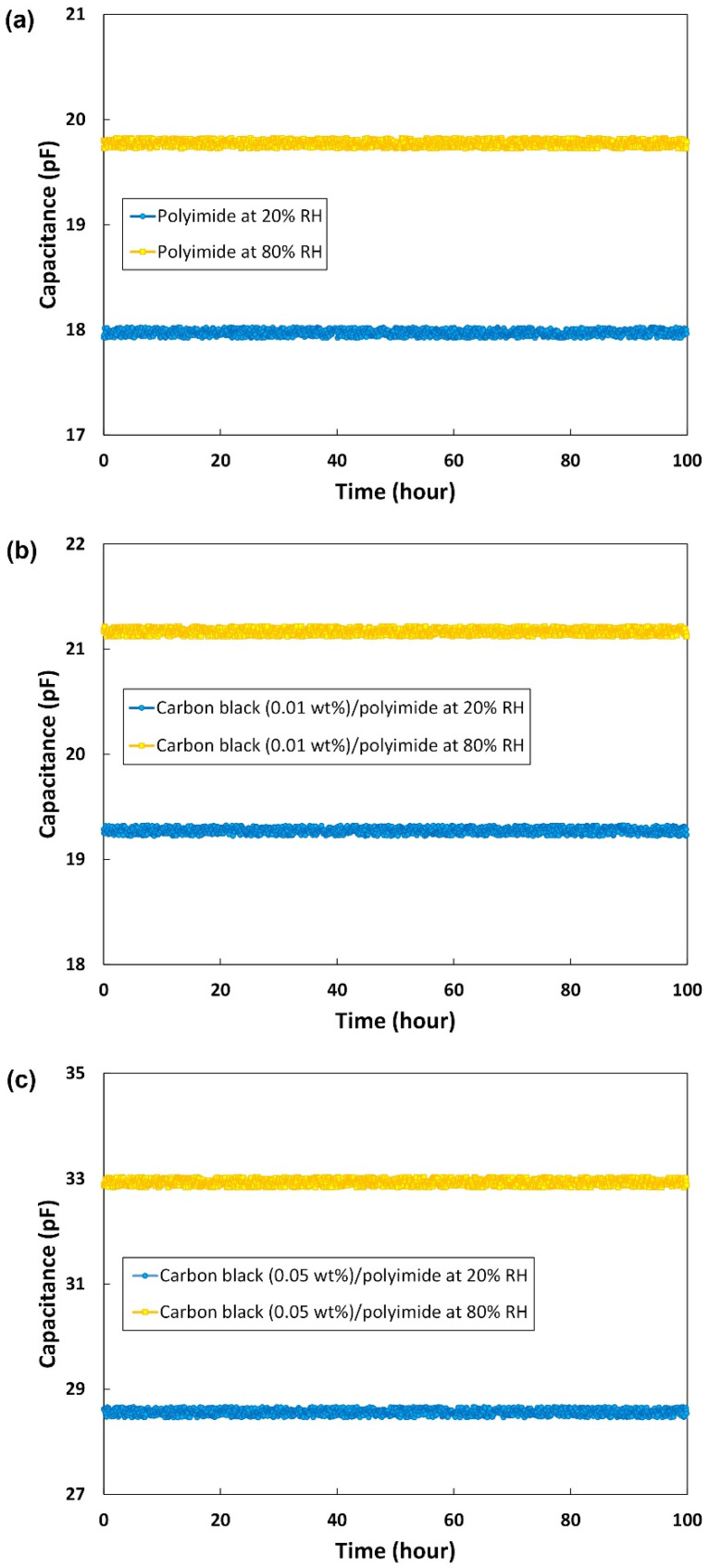
Long-term stability of the humidity sensors with (**a**) pure polyimide, (**b**) carbon black (0.01 wt%)/polyimide, and (**c**) carbon black (0.05 wt%)/polyimide.

To evaluate the long-term stability of the humidity sensor, the capacitance was measured and recorded for 100 h at two different ambient conditions: low humidity level (20% RH) and high humidity level (80% RH). Figure 7a–c show the long-term stability characteristics of the humidity sensors with 0, 0.01, and 0.05 wt% of carbon black, respectively. Because even the humidity sensor made of pure polyimide was very stable, a remarkable effect of the carbon black additive on the long-term stability was not observed. However, as shown in Figure 7, it can be confirmed that the capacitances of all the sensors are maintained very stably with a variation less than ±0.37%. This variation is thought to be mainly due to fluctuations of the humidity generating chamber. Even though the chamber is set at a certain level of humidity, there are always fluctuations, and they affect the output of the sensors. In addition, small temperature fluctuations in the chamber also exist. Because of this problem, our measurement set-up could cause a measurement error of around 1% RH for the three sensors. However, this limit may be improved by the calibration of the equipment or obtaining more data points for averaging.

## 4. Conclusions

In summary, a novel capacitive humidity sensor based on carbon black/polyimide composites has been suggested. There have been efforts to develop resistive-type humidity sensors with carbon-polymer composites of which the conductance is enhanced. However, as far as we know, there have been few attempts to consider the composite as a dielectric for capacitive-type humidity sensors. In this paper, we have successfully demonstrated the possibility of using a carbon black/polyimide composite as a humidity sensing dielectric material to obtain high-performance capacitive humidity sensors. The suggested humidity sensors with polyimide composite filled with tiny amounts of carbon black showed improvements in the sensitivity, hysteresis, and response speed. In the case of the humidity sensor with 0.05 wt% carbon black, a higher sensitivity of 15.21% (0.2535%/% RH) was obtained compared to 9.73% (0.1622%/% RH) for the sensor with pure polyimide, which seems to be due to the presence of the interface between the carbon black additive and the polyimide matrix. In addition, because of the increased contact area with water molecules caused by the carbon black additive, a reduced hysteresis of 1.80% (compared to 2.17%) and a ~10% faster response speed were observed. The long-term stability of the humidity sensors with a small variation was verified as well. Further study exploring humidity sensors with various concentrations of carbon black and the phenomenon at high concentrations to determine the optimal proportion will be the subject of future work.

## Data Availability

Data is contained within the article.
